# Quadrant-Specific Retinal Nerve Fiber Layer Thinning in Hydroxychloroquine Retinal Toxicity: A Controlled OCT-Based Structural and Exposure Analysis in Systemic Lupus Erythematosus

**DOI:** 10.3390/diagnostics16121867

**Published:** 2026-06-16

**Authors:** Aida Geamănu, Ruxandra Angela Pîrvulescu, Diana Tricorache, Nicoleta Anton, Alexandra Diana Vrapciu, Mihaela Oana Romanitan

**Affiliations:** 1 Ophthalmology Department, University of Medicine and Pharmacy “Carol Davila”, 020021 Bucharest, Romania; 2 Ophthalmology Clinic, Emergency University Hospital, 050098 Bucharest, Romania; 3 Department of Ophthalmology, University of Medicine and Pharmacy “Grigore T. Popa”, 700115 Iasi, Romania; 4 Department I, Division of Anatomy, Faculty of Dentistry, University of Medicine and Pharmacy “Carol Davila”, 050474 Bucharest, Romania; 5 Department of Internal Medicine, Södersjukhuset, 11883 Stockholm, Sweden; 6 Karolinska Institutet, Department of Clinical Science and Education by Södersjukhuset, 11883 Stockholm, Sweden

**Keywords:** hydroxychloroquine, retinal toxicity, SLE, structural biomarkers, SD-OCT

## Abstract

**Background:** Hydroxychloroquine (HCQ) is widely used in systemic lupus erythematosus (SLE), yet cumulative exposure may result in progressive retinal toxicity. Structural biomarkers capable of identifying subclinical damage remain incompletely defined. **Methods:** In this cross-sectional controlled study, 60 female SLE patients receiving HCQ for ≥5 years (22 with clinically detectable maculopathy and 38 without) and 30 healthy controls underwent a comprehensive ophthalmologic assessment including spectral-domain optical coherence tomography (SD-OCT). Peripapillary retinal nerve fiber layer (RNFL) and macular thickness parameters were analyzed. Logistic regression and ROC analysis evaluated exposure-related risk. **Results:** Patients with clinically detectable maculopathy demonstrated significant superior and temporal RNFL thinning compared with patients with clinically undetectable maculopathy and controls (*p* ≤ 0.021). Inferior quadrant thinning was detectable in patients without ophthalmoscopic changes, suggesting subclinical neuroaxonal involvement. Parafoveal macular thinning was observed exclusively in the clinically detectable maculopathy group (*p* = 0.041). Cumulative dose >1000 g independently predicted toxicity (OR 3.84; 95% CI 1.72–8.56). The combined structural–exposure model demonstrated strong discrimination (AUC 0.89). **Conclusions:** HCQ-related retinal structural changes may be detectable on OCT in the absence of clinically apparent retinal findings. Our results support the concept of a dose-associated structural continuum in HCQ-related retinal injury, involving both inner retinal neuroaxonal parameters and parafoveal macular alterations. However, the cross-sectional design does not allow determination of the temporal sequence of inner versus outer retinal changes. Further longitudinal studies with combined inner and outer retinal layer-specific analysis are required before these findings can inform modifications to current screening strategies.

## 1. Introduction

Hydroxychloroquine (HCQ) represents a mainstay immunomodulatory therapy in systemic lupus erythematosus (SLE), with established efficacy in reducing disease activity, preventing flares, and improving long-term survival. HCQ retinopathy has emerged as an increasingly recognized complication of long-term therapy, characterized by insidious structural retinal changes that may remain asymptomatic until advanced stages, underscoring the importance of systematic screening and early detection strategies [1]. Consequently, prolonged exposure is common, particularly in female populations requiring chronic disease control. Despite its favorable systemic profile, cumulative HCQ exposure confers a risk of retinal toxicity that may become irreversible once clinically manifest [2].

HCQ retinopathy has classically been defined by parafoveal photoreceptor and retinal pigment epithelium disruption, ultimately progressing toward bull’s-eye maculopathy [3,4]. Recent reviews have emphasized the need for improved structural classification systems integrating modern multimodal imaging findings [5]. Current screening recommendations emphasize weight-adjusted dosing and regular structural evaluation after five years of therapy [2,6,7], and subsequent analyses have demonstrated significant changes in clinical screening practices following implementation of the revised AAO guidelines [1].

Recent international updates have further refined risk stratification approaches across different healthcare systems [8]. Recent real-world cohort data suggest that the prevalence of HCQ retinopathy may be higher than previously estimated. However, clinically detectable toxicity likely reflects a relatively advanced stage of tissue injury, raising concern that earlier structural indicators remain underrecognized. Prior comparative analyses have demonstrated variable sensitivity across screening modalities depending on disease stage [9].

Experimental and imaging evidence suggests that HCQ accumulates within lysosomal compartments [10,11,12], inducing metabolic stress and potentially secondary neuroaxonal compromise. Lysosomal dysfunction and impaired autophagic flux may contribute to secondary neuroaxonal stress within the inner retina [10,11,12]. This pathophysiologic framework supports the hypothesis that inner retinal structures, particularly the peripapillary retinal nerve fiber layer (RNFL), may exhibit measurable alterations prior to overt macular degeneration [13,14]. Histopathological and ultrastructural studies have demonstrated lysosomal alterations and photoreceptor–RPE disruption associated with chronic HCQ exposure [15].

Beyond its well-established therapeutic role in autoimmune disease, HCQ exhibits complex interactions with retinal cellular metabolism that may contribute to progressive structural injury. The drug preferentially accumulates within lysosomes of retinal pigment epithelial (RPE) cells due to its weakly basic properties, leading to intra-lysosomal alkalinization and impairment of enzymatic degradation pathways. This process disrupts normal autophagic flux and interferes with the recycling of photoreceptor outer segments, eventually resulting in metabolic stress within the outer retina. Prolonged lysosomal dysfunction has been associated with the accumulation of lipofuscin-like material, oxidative stress, and progressive cellular degeneration [10,11,12].

While classical descriptions of HCQ retinopathy indicated photoreceptors and RPE damage within the parafoveal region [13], increasing evidence suggests that inner retinal structures may also be affected during earlier stages of toxicity. Experimental models and imaging-based studies have emphasized that neuroaxonal elements of the retina, including ganglion cell bodies and their axons within the retinal nerve fiber layer, may demonstrate measurable structural alterations in the absence of disruption of outer retinal architecture, which become clinically evident. These findings have raised the possibility that early neuroaxonal stress could represent an additional component of the pathophysiologic cascade in drug-related retinal injury [14,16].

Advances in spectral-domain optical coherence tomography (SD-OCT) have enabled high-resolution quantitative assessment of individual retinal layers, facilitating the detection of subtle structural changes that may not be apparent on routine ophthalmoscopic examination. Detailed analysis of peripapillary RNFL thickness and macular layer segmentation has emerged as a valuable approach for identifying early structural indicators in various retinal and neuro-ophthalmic diseases. Within the context of HCQ exposure, systematic evaluation of quadrant-specific RNFL metrics may therefore provide additional insights into early neuroaxonal involvement and help refine existing screening paradigms [5,12,17].

Differential vulnerability between inner and outer retinal layers has been described in various toxic retinopathies [18]. The temporal sequence and clinical relevance of RNFL thinning within the spectrum of HCQ toxicity remain insufficiently characterized. While most prior investigations have focused on macular thickness metrics and outer retinal integrity [16,17,18], quadrant-specific RNFL assessment as an early quantitative biomarker has received comparatively limited attention, particularly in controlled cohorts stratified by clinical retinal findings. Moreover, integration of structural OCT parameters with cumulative exposure variables into multivariate predictive frameworks has been inadequately explored.

Advances in screening strategies have progressively improved the early detection of hydroxychloroquine retinopathy, with comparative analyses demonstrating important differences in sensitivity among available diagnostic modalities and supporting the transition toward objective structural imaging techniques [11]. Despite advances in screening, several challenges still remain regarding early detection and risk stratification of HCQ retinal toxicity [19]. The potential role of inner retinal structural biomarkers, including quadrant-specific peripapillary RNFL alterations, has not been fully characterized in controlled cohorts of HCQ-treated patients. Furthermore, the integration of quantitative OCT-derived structural parameters with cumulative drug exposure metrics remains insufficiently explored.

The present study therefore aimed to evaluate quadrant-specific RNFL alterations and macular thickness changes in patients with systemic lupus erythematosus receiving long-term HCQ therapy and investigate the relationship between structural OCT biomarkers and cumulative HCQ exposure.

## 2. Materials and Methods

### 2.1. Study Design and Participants

This cross-sectional controlled study included 60 female patients diagnosed with SLE undergoing HCQ therapy for at least five years. Patients were stratified into the following groups:

1. **Group I (*n* = 22)** (clinically detectable maculopathy group): patients with clinically detectable HCQ retinopathy based on multimodal assessment (fundus examination, fundus autofluorescence, and visual field abnormalities consistent with toxicity).
2. **Group II (*n* = 38)** (clinically undetectable maculopathy group): patients without clinically detectable retinal toxicity on multimodal evaluation.

A control group of 30 age-comparable healthy subjects without systemic or ocular pathology was included. Group classification was based on multimodal assessment rather than clinical retinal findings alone. Classification was performed at the patient level and did not require bilateral involvement; the presence of characteristic findings in at least one eye was sufficient for group allocation. For statistical analysis, a single eye per subject was included to ensure independence of observations.

Exclusion criteria included diabetes mellitus, glaucoma, high myopia (>6 diopters), previous ocular surgery affecting the posterior pole, and retinal diseases unrelated to HCQ exposure.

The selection of variables for the regression model was based on clinical relevance. The cumulative dose threshold of 1000 g was chosen based on previously reported risk stratification data.

Post hoc pairwise comparisons were performed where appropriate, and results were interpreted in the context of multiple comparisons given the exploratory nature of the analysis.

Weight-adjusted dose (mg/kg/day) was calculated as daily dose divided by ideal body weight (kg), using standard clinical formulas.

The cumulative dose was calculated as the product of daily dose and duration of therapy.

### 2.2. Ophthalmologic Assessment

All participants underwent best-corrected visual acuity (BCVA), intraocular pressure measurement, slit-lamp exam, dilated fundus examination, fundus autofluorescence, 10° automated visual field testing, and spectral-domain optical coherence tomography (SD-OCT).

Peripapillary RNFL thickness (global and quadrant-specific) and macular thickness parameters (central, parafoveal, and perifoveal) were recorded. SD-OCT measurements were considered primary structural biomarkers.

### 2.3. OCT Acquisition and Image Quality Control

SD-OCT imaging was performed using a Cirrus HD-OCT system (Carl Zeiss Meditec, Dublin, CA, USA) according to a standardized acquisition protocol. Peripapillary retinal nerve fiber layer (RNFL) measurements were obtained using the Optic Disc Cube 200 × 200 scan protocol, which generates a three-dimensional dataset centered on the optic nerve head and provides automated RNFL thickness measurements along a 3.46 mm diameter peripapillary circle. Macular thickness parameters were obtained using the Macular Cube 512 × 128 scan protocol, allowing quantitative evaluation of central, parafoveal, and perifoveal retinal thickness within the Early Treatment Diabetic Retinopathy Study (ETDRS) grid.

OCT segmentation was reviewed by experienced operators to ensure accuracy of layer identification under standardized conditions after pupillary dilation. Image quality was assessed based on signal strength and the absence of motion artifacts. Only scans with signal strength ≥7 and without segmentation errors were included in the final analysis. In cases where image quality was suboptimal, the scan was repeated to ensure reliable measurements.

Automated segmentation provided global and quadrant-specific RNFL thickness values (superior, inferior, nasal, and temporal), as well as macular thickness parameters including central foveal thickness, mean parafoveal thickness, and mean perifoveal thickness. All OCT datasets were reviewed to confirm correct layer segmentation before inclusion in the analysis.

### 2.4. Exposure Variables

HCQ exposure was quantified using:

1. Daily dose (mg/day);
2. Weight-adjusted dose (mg/kg/day);
3. Duration of therapy (years);
4. Cumulative dose (grams).

Weight-adjusted dosing was calculated according to ideal body weight formulas, as follows: 48.67 kg + 1.65 kg/inch greater than 5 feet (1.52 m); 1 inch = 2.54 cm [2].

### 2.5. Statistical Analysis

For each participant, a single eye was included in the analysis. The eye with the most pronounced structural alterations was selected in order to avoid inter-eye correlation and ensure independence of observations.

Normality was assessed using the Shapiro–Wilk test.

Continuous variables are expressed as mean ± standard deviation (SD). Between-group comparisons were performed using:

1. One-way ANOVA for three-group comparisons (with post hoc Bonferroni correction when applicable);
2. Welch’s *t*-test for two-group comparisons with unequal variances.

Given the exploratory nature of quadrant-specific analyses, *p*-values were interpreted cautiously.

Multivariate logistic regression analysis was conducted to identify independent predictors of retinal toxicity. Considering the limited number of events (*n* = 22), the model was restricted to clinically relevant covariates to minimize overfitting. Odds ratios (ORs) with 95% confidence intervals (CIs) were reported.

Receiver operating characteristic (ROC) analysis was performed to evaluate discriminatory ability. The area under the curve (AUC) was calculated. A *p*-value < 0.05 was considered statistically significant.

### 2.6. Ethical Considerations

The study protocol was reviewed and approved by the Ethics Committee of “Carol Davila” University of Medicine and Pharmacy, Bucharest, Romania (Approval No. 84/3 February 2025), prior to patient enrollment. The study adhered to the tenets of the Declaration of Helsinki. Written informed consent was obtained from all participants.

No personal identifying information was included, and all data was anonymized.

## 3. Results

Baseline demographic and exposure characteristics are summarized in Table 1.

Patients with clinically detectable maculopathy demonstrated significantly longer duration of HCQ therapy and higher cumulative exposure compared with patients with clinically undetectable maculopathy.

### 3.1. RNFL Analysis (Table 2)

Significant thinning was observed in the superior and temporal quadrants in Group I compared with both Group II and controls (*p* ≤ 0.021). Inferior quadrant thinning was detectable in Group II despite the absence of clinically apparent retinal changes (*p* = 0.001), suggesting subclinical neuroaxonal involvement. Global RNFL thickness was significantly reduced in the clinically detectable maculopathy group (*p* < 0.001).

**Table 2:** RNFL thickness by quadrant (µm).

Quadrant	Group I	Group II	Controls	*p*-Value
**Global RNFL**	87.3 ± 9.5	92.8 ± 8.7	98.4 ± 7.9	<0.001
**Superior**	103.6 ± 14.2	111.4 ± 13.6	118.9 ± 12.8	0.021
**Inferior**	99.8 ± 12.7	104.3 ± 11.9	116.7 ± 13.4	0.001
**Nasal**	69.4 ± 9.8	73.8 ± 8.9	75.6 ± 7.5	0.087
**Temporal**	58.6 ± 7.3	64.2 ± 6.8	71.5 ± 6.2	0.001

Pairwise comparisons showed that differences between the clinically detectable maculopathy group and controls were most consistent for global RNFL thickness and for the inferior and temporal RNFL quadrants and slightly less robust for superior RNFL. Nasal RNFL differences did not reach statistical significance. These findings should be interpreted cautiously, given the exploratory nature of the analysis and the limited sample size.

The magnitude of the global RNFL difference was as follows: the mean global RNFL thickness was 11.1 µm lower in the clinically detectable maculopathy group than in controls, corresponding to an approximately 11.3% relative reduction compared with the control mean.

### 3.2. Macular Analysis (Table 3)

Central foveal thickness remained preserved across groups. Parafoveal thinning was significant in Group I (*p* = 0.041), consistent with the classical parafoveal pattern of HCQ toxicity. Perifoveal changes did not reach statistical significance.

**Table 3:** Macular thickness analysis (µm).

Parameter	Group I	Group II	Controls	*p*-Value
**Central foveal thickness**	248.6 ± 18.4	251.9 ± 16.7	253.1 ± 15.9	0.62
**Parafoveal thickness (mean)**	287.3 ± 14.6	298.5 ± 15.2	304.7 ± 13.8	0.041
**Perifoveal thickness (mean)**	274.8 ± 16.9	281.6 ± 17.4	285.2 ± 14.3	0.072

Pairwise comparisons showed that parafoveal thickness differed between the clinically detectable maculopathy group and controls, whereas central foveal thickness did not differ significantly. Perifoveal thickness showed a less robust between-group difference relative to conventional statistical significance. These findings are interpreted as structural associations rather than evidence of a temporal sequence of macular involvement.

For the main structural outcomes, mean values were lower in the clinically detectable maculopathy group than in controls. The mean differences were −11.1 µm for global RNFL thickness (95% CI: −16.1 to −6.1), −16.9 µm for inferior RNFL thickness (95% CI: −24.2 to −9.6), −12.9 µm for temporal RNFL thickness (95% CI: −16.8 to −9.0), and −17.4 µm for parafoveal macular thickness (95% CI: −25.5 to −9.3). These confidence intervals support the presence of measurable structural thinning in the clinically detectable maculopathy group compared with controls, particularly at the level of the global, inferior, and temporal RNFL and parafoveal macular thickness.

### 3.3. Risk Modeling

Cumulative dose >1000 g emerged as an independent predictor of toxicity (OR 3.84; 95% CI 1.72–8.56). The combined structural–exposure model demonstrated good discriminatory performance, with an AUC of 0.89 (95% CI: 0.79–0.99), although this finding should be interpreted cautiously in the context of the limited sample size.

## 4. Discussion

Our controlled multimodal evaluation supports a dose-dependent structural continuum of hydroxychloroquine-related retinal injury in SLE. Importantly, quantitative SD-OCT abnormalities were detectable in patients both with and without clinically apparent retinal changes, supporting the role of OCT in the structural assessment of subclinical retinal involvement [13,20,21]. Recent studies have confirmed that structural imaging enables the detection of preclinical retinal alterations in the absence of symptomatic toxicity [22,23].

**Figure 1:**
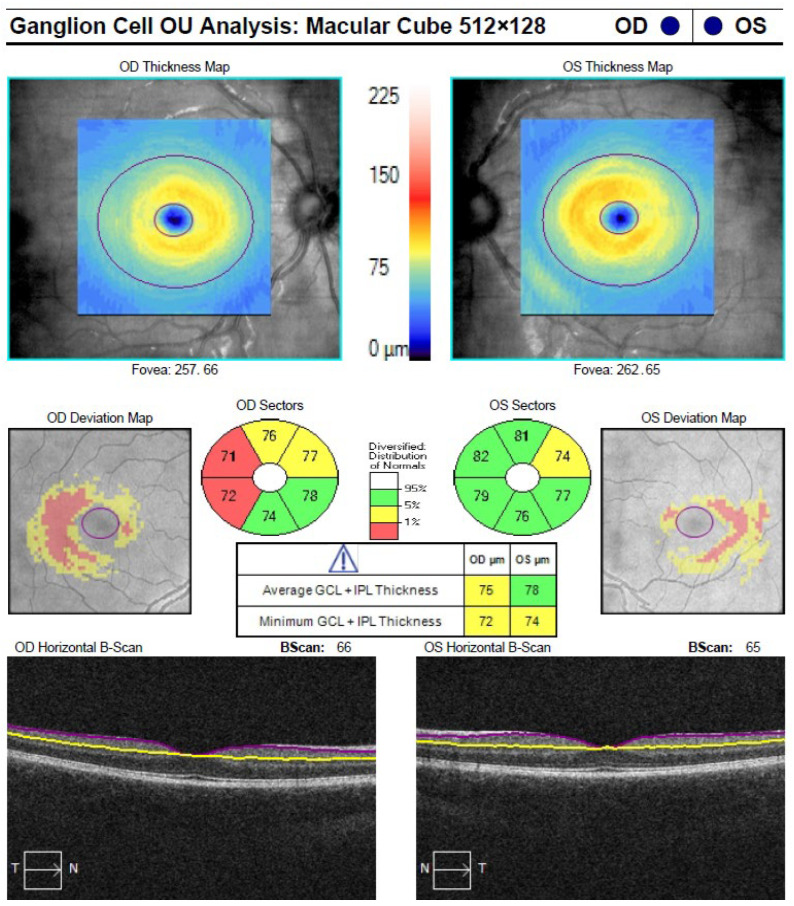
Ganglion cell analysis in a patient from Group II.

**Figure 2:**
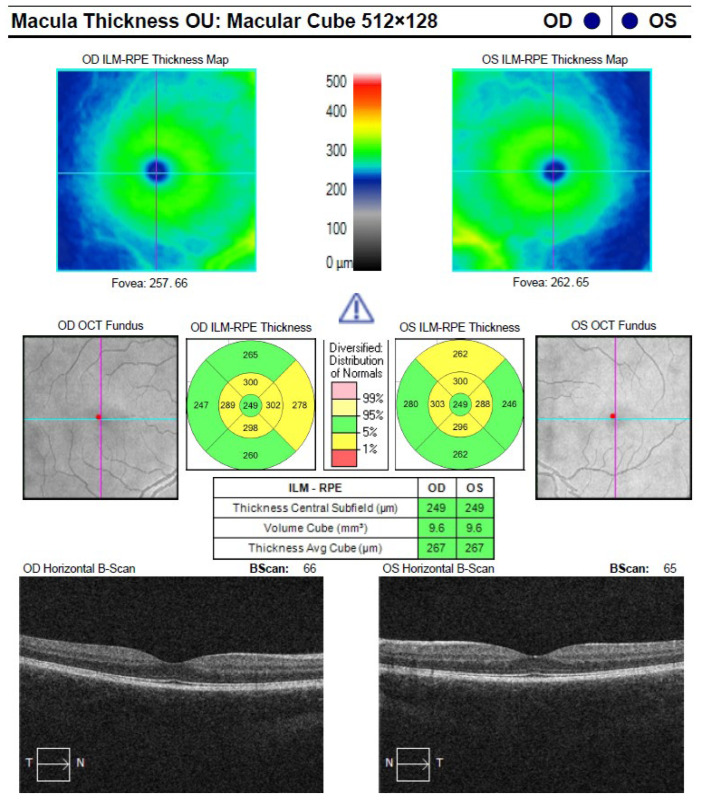
Macular thickness map—ILM-RPE of a patient from Group II.

**Figure 3:**
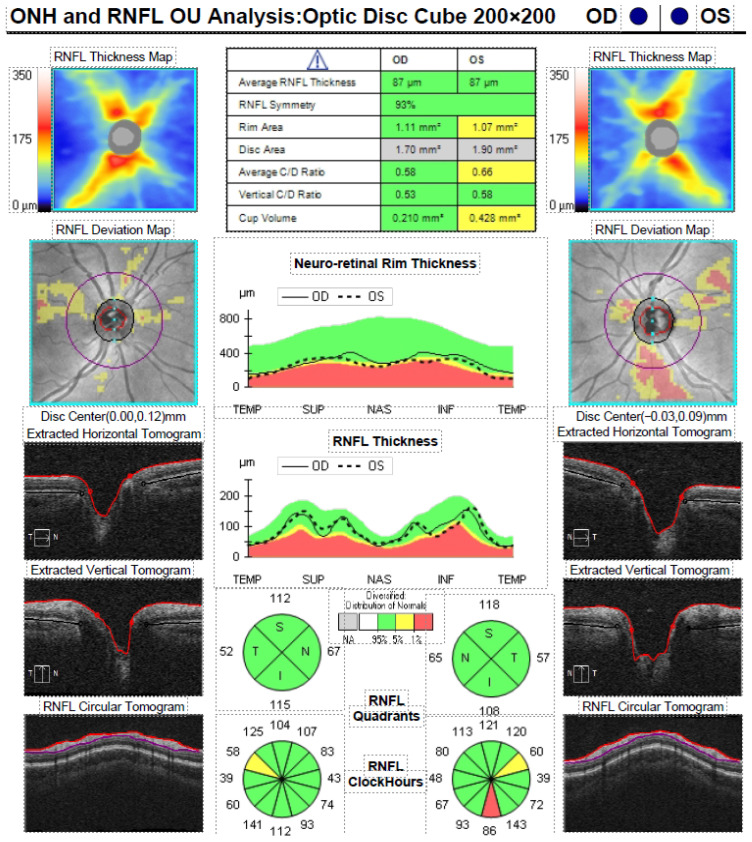
Peripapillary RNFL analysis in a patient from Group II.

We exemplify this aspect in Figure 1, Figure 2 and Figure 3, representing OCT examinations from a 17-year-old female patient with systemic lupus erythematosus who had been receiving hydroxychloroquine 400 mg/day for more than 5 years; the patient belongs to Group II (the group of patients without clinically apparent retinal changes). Spectral-domain optical coherence tomography (SD-OCT) performed with a Cirrus HD-OCT (Carl Zeiss Meditec) demonstrated in this case preserved overall macular architecture with subtle but topographically localized inner retinal alterations. This case should be interpreted as illustrative rather than representative of cohort-level findings.

Ganglion cell–inner plexiform layer (GCL–IPL) analysis (Figure 1) revealed localized parafoveal deviations, most pronounced in the right eye. Sectoral analysis demonstrated two abnormal and two borderline parafoveal sectors in the right eye, predominantly within the temporal–inferotemporal hemiring, while the left eye showed only minimal sectoral deviation. The deviation map (Figure 2) displayed an annular parafoveal pattern of localized reduction in full retinal thickness centered approximately 2–4° from the fovea, a distribution that corresponds topographically to the region classically vulnerable to hydroxychloroquine exposure. The observed changes reflect full retinal thickness rather than isolated inner retinal layer alterations.

On peripapillary RNFL analysis, clock-hour analysis showed focal sectoral irregularities, including one abnormal clock-hour sector and one borderline sector in the left eye, suggesting localized neuroaxonal variation not reflected in quadrant averages (Figure 3).

The structural OCT features observed in the illustrative case are consistent with the broader pattern detected in our quantitative analysis. While global RNFL and quadrant metrics remained within normal limits, focal clock-hour deviations were present, suggesting spatially restricted neuroaxonal variation not captured by quadrant averages. This pattern mirrors the cohort-level findings, where subtle quadrant-specific RNFL differences were detectable despite preserved global RNFL thickness. In combination with the localized parafoveal ganglion cell thinning observed on GCL–IPL analysis, these findings support the hypothesis that layer-specific inner retinal alterations may occur along a structural continuum of hydroxychloroquine exposure in the absence of clinically apparent macular toxicity.

Overall, peripapillary RNFL analysis revealed a spatially selective pattern: superior and temporal thinning characterized the clinically detectable maculopathy group. Age-related retinal thinning may have contributed to the observed differences and should be considered a potential confounding factor when interpreting these findings.

Quadrant-specific RNFL thinning was observed not only in patients with clinically apparent toxicity but also in those without clinically apparent retinal changes, suggesting subclinical neuroaxonal involvement within the spectrum of HCQ-related retinal injury. Emerging OCT angiography studies suggest that inner retinal and microvascular alterations may occur even in patients without overt toxicity [24].

Temporal RNFL vulnerability plausibly reflects early involvement of the papillomacular bundle [10,11], while inferior RNFL changes observed in the clinically undetectable maculopathy group suggest that neuroaxonal alterations may coexist with, or be detectable in the absence of, overt RPE/photoreceptor abnormalities on routine examination. Similar peripapillary alterations have been reported in drug-related retinal injury models [13,14].

The identification of localized RNFL alterations in the present study, in patients without clinically detectable toxicity, also raises important considerations regarding current screening paradigms. Traditional descriptions of HCQ retinopathy have mainly focused on parafoveal photoreceptor and retinal pigment epithelium disruption, which eventually culminates in classical bull’s-eye maculopathy [13,25,26]. However, advances in high-resolution retinal imaging increasingly suggest that structural alterations may occur across multiple retinal layers in the absence of overt macular damage and become clinically apparent [14,16,18]. In this context, neuroaxonal stress affecting ganglion cells and their axons may represent an additional structural component of hydroxychloroquine-related retinal injury.

Spectral-domain OCT provides the opportunity to evaluate these subtle structural changes in vivo through quantitative assessment of both macular and peripapillary retinal layers. While macular thickness metrics remain essential for detecting established HCQ toxicity, detailed analysis of peripapillary RNFL thickness may offer additional information regarding early neuronal involvement. To be more specific, quadrant-specific RNFL evaluation may reveal spatially selective patterns of structural alteration that are not evident when relying only on global RNFL averages or macular thickness measurements [26,27,28].

From a clinical perspective, RNFL analysis may provide complementary structural information during longitudinal follow-up of patients receiving long-term HCQ therapy. However, current screening guidelines remain centered on macular structural and functional testing. Additional longitudinal studies are required to determine whether quadrant-specific RNFL alterations have independent predictive value and whether they should be incorporated into future screening strategies [17,29,30].

Macular metrics complemented the neuroaxonal signal identified on RNFL analysis: central foveal thickness remained preserved, whereas parafoveal thinning in the clinically detectable maculopathy group aligned with the classical parafoveal predilection of hydroxychloroquine retinopathy. An important consideration raised by the present findings is the relative temporal relationship between inner and outer retinal structural alterations in hydroxychloroquine-related toxicity. While classical descriptions emphasize early parafoveal photoreceptor and retinal pigment epithelium disruption, our results suggest that measurable inner retinal changes, particularly at the level of the peripapillary RNFL and ganglion cell complex, may be detectable in patients without clinically apparent macular involvement.

However, given the cross-sectional design of the study, no definitive conclusions can be drawn regarding the chronological sequence of these changes. It therefore remains unclear whether inner retinal alterations precede, accompany, or represent a parallel manifestation of outer retinal injury.

From a clinical perspective, outer retinal biomarkers continue to represent the cornerstone of current screening strategies, as reflected in existing guidelines. While our findings support the concept of a structural continuum, further longitudinal studies integrating detailed layer-specific analysis are required before such observations can inform modifications of screening protocols.

Outer retinal alterations, particularly in the inferior parafoveal region, have been reported as early markers of HCQ toxicity and may represent a complementary structural biomarker. A detailed layer-specific comparison between inner and outer retinal biomarkers was not the primary objective of the present cross-sectional analysis and therefore could not be fully addressed within the current study design. Together, these findings support a dual-structure framework in which neuroaxonal involvement (RNFL) coexists with parafoveal macular susceptibility [16,23,31].

From an exposure perspective, a cumulative dose >1000 g [11,14,32] emerged as an independent predictor of toxicity (OR 3.84; 95% CI 1.72–8.56), and the combined structural–exposure model demonstrated strong discrimination (AUC 0.89). This distinction may be particularly relevant in patients exceeding cumulative dose thresholds, where earlier structural detection could theoretically refine risk stratification. Dose-dependent risk relationships have been previously established in large cohort analyses [14,32,33]. Variability in individual susceptibility to hydroxychloroquine retinopathy has been further supported by studies demonstrating an association between systemic drug exposure levels and toxicity risk, suggesting that factors beyond weight-adjusted dosing may influence retinal vulnerability [31]. While the present report emphasizes overall discriminatory performance rather than a single prespecified cut-off, the observed AUC supports clinically meaningful risk stratification when quantitative OCT is integrated with exposure variables. Sequential structural analyses have suggested that hydroxychloroquine retinopathy is associated with progressive structural alterations, with subtle structural abnormalities detectable in the absence of the development of characteristic clinical manifestations, further supporting a progressive structural model of hydroxychloroquine-related retinal injury [34].

The structural–functional dissociation observed in our cohort, with preserved BCVA and functional parameters despite OCT abnormalities, is clinically relevant [12,15]. It indicates that reliance on functional testing alone may underestimate early toxicity, and it supports guideline-concordant OCT-centered surveillance, particularly in individuals exceeding recommended weight-adjusted dosing thresholds or with reduced renal function. This phenomenon has been consistently described in early hydroxychloroquine toxicity and other retinal toxicities [13]. Recent reports suggest that toxicity may occur despite adherence to recommended weight-adjusted dosing, supporting individualized monitoring strategies [14].

Clinical implications include (i) prioritizing weight-adjusted dosing, (ii) incorporating quadrant-specific RNFL metrics into longitudinal follow-up, and (iii) emphasizing cumulative exposure tracking. These measures may support more comprehensive risk characterization during long-term HCQ therapy, although their role in modifying screening intervals requires prospective longitudinal validation. Multimodal imaging studies have demonstrated that hydroxychloroquine retinopathy is characterized by distinct structural patterns detectable across multiple retinal layers, reinforcing the role of quantitative imaging in identifying early disease manifestations [33]. Progressive retinal damage has been reported even after discontinuation of HCQ therapy, emphasizing the importance of early structural detection [5,32].

## 5. Limitations and Strengths

This study has several limitations.

First, its cross-sectional design precludes assessment of temporal progression and does not allow definitive conclusions regarding the sequence of structural changes observed in HCQ-related retinal injury.

Second, the relatively limited number of toxicity cases may have constrained multivariate modeling despite restrictions to clinically relevant covariates.

Third, the cohort consisted of female patients, reflecting the epidemiology of systemic lupus erythematosus but potentially limiting broader generalizability. Finally, advanced functional testing modalities such as multifocal electroretinography or OCT angiography were not available, preventing a comprehensive structural–functional correlation.

Fourth, the selection of the more affected eye was intended to capture clinically relevant structural changes and may have introduced a degree of selection bias by potentially overestimating structural differences between groups. Formal masking of the OCT image review was not performed, which may represent a potential source of assessment bias.

Last, but not least, the significant age difference between groups represents a potential confounding factor, as age-related retinal thinning may have influenced RNFL and macular thickness measurements. Formal age-adjusted analyses were not performed in the present study.

Despite these limitations, the controlled design including HCQ-treated patients with and without toxicity, together with an age-comparable healthy control group, enabled characterization of structural alterations across different stages of retinal involvement. The integration of quantitative OCT-derived metrics with cumulative exposure assessment provides clinically relevant insights that reflect real-world screening conditions and supports the internal consistency of the observed findings.

## 6. Conclusions

In conclusion, controlled OCT-based structural analysis in HCQ-treated SLE patients demonstrates distinct patterns of peripapillary RNFL thinning and parafoveal macular alteration associated with cumulative exposure. Quadrant-specific RNFL changes were detectable even in the absence of clinically detectable toxicity, supporting the concept of a dose-dependent structural continuum in HCQ-related retinal injury.

These findings suggest that quadrant-specific RNFL analysis may represent a complementary structural parameter associated with subclinical HCQ-related retinal involvement. Integration of quantitative OCT-derived metrics with cumulative dose assessment may enhance structural characterization and risk stratification in long-term HCQ therapy. However, the relative temporal sequence of inner retinal neuroaxonal changes and outer retinal macular alterations cannot be established from the present cross-sectional data. Further longitudinal studies incorporating combined inner and outer retinal layer-specific analysis are required to determine the predictive value of these biomarkers and to assess whether they should influence future screening strategies.

## Figures and Tables

**Table 1:** Baseline characteristics of study participants.

Parameter	Group I (Clinically Detectable Maculopathy, *n* = 22)	Group II (Clinically Undetectable Maculopathy, *n* = 38)	Controls (*n* = 30)	*p*-Value
**Age (years)**	54.9 ± 8.2	46.4 ± 10.4	48.6 ± 11.6	0.011
**Duration of HCQ therapy (years)**	11.2 ± 3.9	8.4 ± 3.0	—	0.006
**Daily dose (mg/day)**	400.0 ± 0.0	384.2 ± 54.7	—	0.083
**Weight-adjusted dose (mg/kg/day)**	5.0 ± 1.4	5.3 ± 1.7	—	0.405
**Cumulative dose (g)**	1665.7 ± 613.9	1229.5 ± 438.2	—	0.006

## Data Availability

The data presented in this study are available from the corresponding author upon reasonable request. The data are not publicly available due to privacy and ethical restrictions.
